# Saffron (*Crocus sativus* L.) Flower Water Extract Disrupts the Cecal Microbiome, Brush Border Membrane Functionality, and Morphology In Vivo (*Gallus gallus*)

**DOI:** 10.3390/nu14010220

**Published:** 2022-01-05

**Authors:** Nikita Agarwal, Nikolai Kolba, YeonJin Jung, Jacquelyn Cheng, Elad Tako

**Affiliations:** Department of Food Science, Cornell University, Ithaca, NY 14850, USA; na494@cornell.edu (N.A.); nk598@cornell.edu (N.K.); yj354@cornell.edu (Y.J.); jyc53@cornell.edu (J.C.)

**Keywords:** *Crocus sativus*, polyphenols, intestine, microbiome, gene expression, saffron, petal

## Abstract

Saffron (*Crocus sativus* L.) is known as the most expensive spice. *C. sativus* dried red stigmas, called threads, are used for culinary, cosmetic, and medicinal purposes. The rest of the flower is often discarded, but is now being used in teas, as coloring agents, and fodder. Previous studies have attributed antioxidant, anti-inflammatory, hepatoprotective, neuroprotective, anti-depressant, and anticancer properties to *C. sativus* floral bio-residues. The aim of this study is to assess *C. sativus* flower water extract (CFWE) for its effects on hemoglobin, brush boarder membrane (BBM) functionality, morphology, intestinal gene expression, and cecal microbiome in vivo (*Gallus gallus*), a clinically validated model. For this, *Gallus gallus* eggs were divided into six treatment groups (non-injected, 18 Ω H_2_O, 1% CFWE, 2% CFWE, 5% CFWE, and 10% CFWE) with *n*~10 for each group. On day 17 of incubation, 1 mL of the extracts/control were administered in the amnion of the eggs. The amniotic fluid along with the administered extracts are orally consumed by the developing embryo over the course of the next few days. On day 21, the hatchlings were euthanized, the blood, duodenum, and cecum were harvested for assessment. The results showed a significant dose-dependent decrease in hemoglobin concentration, villus surface area, goblet cell number, and diameter. Furthermore, we observed a significant increase in Paneth cell number and Mucin 2 (MUC2) gene expression proportional to the increase in CFWE concentration. Additionally, the cecum microbiome analysis revealed *C. sativus* flower water extract altered the bacterial populations. There was a significant dose-dependent reduction in *Lactobacillus* and *Clostridium* sp., suggesting an antibacterial effect of the extract on the gut in the given model. These results suggest that the dietary consumption of *C. sativus* flower may have negative effects on BBM functionality, morphology, mineral absorption, microbial populations, and iron status.

## 1. Introduction

*Crocus sativus* L. is a stemless perennial plant that belongs to the Iridaceae family. It is commonly cultivated in Iran, Spain, Morocco, Turkey, India, Greece, and Italy for its stigma [1,2]. The flowers comprise of six purple tepals (petal and sepals are fused), a white style surrounded by three yellow stamens and a red stigma divided into three threads [3,4]. Saffron is the dried stigma of the plant which is hand-picked and the rest of the flower (~95% by weight) is either thrown away, used as fodder, as food additive, or in beverages. The floral bio-residues have limited literature describing their health benefits.

Saffron (stigma), although, is reported to have numerous pharmacological benefits such as antioxidant [5], anti-inflammatory [6], hepatoprotective [7], neuroprotective [8], antidepressant [9], and anti-tumorigenic [8] activity among others. Most of these therapeutic effects are accredited to three major bioactive compounds: crocin (responsible for saffron’s color), picrocrocin (its bitter taste), and safranal (its aroma) [10]. These compounds can be found in the tepals as well [1,4,11,12,13]. Additionally, the floral bio-residues have numerous flavonoids including kaempferol (84% of total flavonols), quercetin, delphinidin, petunidin, and malvidin [4]. Anthocyanins are water-soluble plant pigments that have been reported to provide beneficial effects on intestinal health, specifically, improving intestinal microbiome, goblet cell number, villi surface area, and short chain fatty acid production [14]. Kaempferol, on its own, has been shown to improve intestinal barrier function, improve goblet cell function, and reduce the expression of inflammatory markers such as interleukin-8 (IL-8) [15,16]. As for the nutritional composition, floral bio-residues are found to have moisture (~10 g), ash (~8.5 g), proteins (~9.5 g), lipids (~2.8), and carbohydrates, including fiber, (~60 g per 100 g dry weight) providing approximately 300 kcal per 100 g dry weight [3]. The presence of saponins, terpenoids, tannins, and steroids have also been reported [17,18].

*C. sativus* floral bio-residues and its isolated bioactive compounds have been investigated previously via in-vitro, in-vivo, and human studies. In general, some evidence supports its effect as an antioxidant [11,19,20,21,22], anti-inflammatory agent [2,11,23,24,25], lipid lowering potential [22,26], hepatoprotective [20], and antidepressant activity [27,28,29]. With this study, we aim to investigate the effect of *C. sativus* flower water extract (CFWE) on intestinal brush border membrane functionality, morphology, iron status, intestinal gene expression, and cecal microbiome. To the best of our knowledge, CFWE has previously not been investigated on these parameters.

Natural products such as anthocyanins are not readily absorbed but are transformed by the gut microbiome [30]. Therefore, the consumption of these phytocompounds have the potential to alter the diversity and composition of the commensal bacterial species [31]. Recent literature has suggested the link between the microbiome and disease conditions—such as inflammatory bowel disease, type 2 diabetes, cardiovascular diseases, cancer, and obesity [32]. So far, the effect of *C. sativus* floral bio-residues have not been evaluated on the microbiome. With this study, we aim to get a cursory glance at the changes associated with the dietary consumption of CFWE at the genus level through 16s rDNA analysis.

For this study, we utilized *Gallus gallus*, in-vivo administration, a well-established and widely accepted model for the assessment of bioactive compounds [33,34,35,36,37,38,39,40,41,42,43,44]. It has been previously demonstrated that the *Gallus gallus* model is an effective tool in evaluating mineral deficiencies [33,37,45,46]. The amino acid sequence of transporter genes (Fe and Zn) is 85% homologous to humans. Furthermore, the cecal gut microbiome of *Gallus gallus* at the phylum level is very similar to that observed in the human gut. Thus, the changes observed here can potentially be used to predict the changes that would be expected clinically. Here, we administer 1 mL of water-soluble extracts of different concentrations in the amnion of the *Gallus gallus* egg. The embryo orally consumes the amniotic fluid before hatch. The physiological changes observed in the hatchlings give an indication of the effects CFWE may have. As most flavonoids (including anthocyanins) and crocin in *C. sativus* floral bio-residues are water soluble, we hypothesize based on existing literature that CFWE would have an overall beneficial effect on the parameters assessed on gut health.

## 2. Materials and Methods

### 2.1. Extract Preparation

Saffron flower bio-residues—i.e., *C. sativus* flower without its stigma—acquired from a New York State Farmer were utilized in this study. The extraction was performed as previously described [40,45]. Briefly, fresh flowers were frozen at −80 °C and powdered. The powder was dissolved in 50 g/L distilled water for 60 min at 60 °C. The particulate matter was removed by centrifugation at 3000× *g* for 25 min at 4 °C. The supernatant remaining was dialyzed exhaustively for 48 h against distilled water. The dialysate (MWCO 12–14 kDa) collected was lyophilized which resulted in a light purple powder.

### 2.2. Analysis of Polyphenols

#### 2.2.1. Polyphenol Extraction

A solution of methanol: water (50:50 *v*/*v*) was prepared and 5 mL of this was added to 500 mg of saffron flower water extract (prepared in 2.1). The extract was vortexed for 1 min then sonicated at room temperature for 20 min. The extracts were vortexed again and placed at room temperature for 60 min on a compact digital Rocker (Labnet International, Inc., Edison, NJ, USA). After which they were centrifuged at 4000× *g* for 15 min. Supernatants were filtered using a 0.2 µm Teflon™ syringe filter. The extracts were stored until analysis at −20 °C.

#### 2.2.2. Liquid Chromatography-Mass Spectroscopy (LC-MS)

The standards and extracts were analyzed as described earlier [39]. In short, UHPLC (Thermo Vanquish UHPLC C18 System, Waltham, MA, USA) coupled to an Advion expressionL^®^ compact mass spectrometer (CMS; Advion Inc., Ithaca, NY, USA). Compound Discoverer™ software 3.1 (Thermo scientific, Waltham, MA, USA) was used to control the LC and CMS instrumentation and data acquisition. The *m*/*z* ratio and LC retention time was used to identify and confirm individual polyphenols against authentic standards. Polyphenol standard curves for flavonoids were derived from integrated areas under UV absorption peaks with five replicates.

### 2.3. Animals and Study Design

About 60 fertile Cornish-cross broiler eggs were purchased from a commercial hatchery (Moyer’s chicks, Quakertown, PA, USA). The eggs were incubated until hatch at the Cornell University Animal Science poultry farm. The protocol carrier out was approved by Cornell University Institutional Animal Care and Use committee. The protocols were carried out in accordance with the relevant regulations and guidelines. For the in vivo administration, 60 viable (fertile) eggs were weighed and randomly distributed into 6 groups (*n* = 10). On day 17 of incubation, candling was used to confirm fertilization and determine the injection spot. The spot alone was then sanitized followed by a 1 mL injected of the prepared water extracts and control (H_2_O). The six treatments were (1) no injection, (2) 18 Ω H_2_O, (3) 1% CFWE, (4) 2% CFWE, (5) 5% CFWE, and (6) 10% CFWE. After the intra-amniotic administrated, the injection spot was sealed with cellophane tape and the eggs were incubated treatment-wise until hatch (day 21) [33,42]. On the day of the hatch, the birds were euthanized in a CO_2_ chamber and the sample collection was carried out. The blood, duodenum, and cecum were removed and immediately placed in liquid nitrogen (except blood which was placed on ice) temporarily. The samples were then transferred to a −80 °C incubator until analysis.

### 2.4. Blood Analysis and Hb Measurements

The blood was collected from the heart and put in micro-hematocrit heparinized capillary tubes (Fisher Scientific, Waltham, MA, USA). The blood was shaken in the heparinized tubed to prevent coagulation. A spectrophotometer (QuantiChrom™ Hemoglobin Assay DIHB-250, BioAssay Systems, Hayward, CA, USA) was used to determine the blood Hb concentration on the same day of the hatch. The kit manufacturer’s instructions were followed.

### 2.5. Gene Expression Analysis

#### 2.5.1. Isolation of Total RNA from the Duodenum

About 30 mg of duodenum (*n* = 5), that was cut near the duodenal loop, was weighed and the total RNA was extracted according to the manufacturer’s protocol using the Qiagen RNeasy Mini Kit (RNeasy Mini Kit, Qiagen Inc., Valencia, CA, USA). All steps were carried out in an RNase free environment [45,46]. In short, the duodenum tissues were homogenized in buffer RLT^®^, containing β-mercaptoethanol. The homogenate was centrifuged at 8000× *g* for 3 min and the supernatant was transferred to another tube containing 70% ethanol. Each sample (700 μL) was put in a RNeasy mini column, centrifuged at 8000× *g* for 15 s, the flow through was discarded. Then, the RNeasy columns were transferred to new 2 mL collections tubes. The RPE^®^ buffer (500 μL) from the kit was pipetted into the RNeasy column and centrifuged at 8000× *g* for 2 min. The RNA eluted in 50 μL of RNase free water and quantified at absorbance A 260/280. RNA integrity of the 18S ribosomal region was verified by 1.5% agarose gel electrophoresis stained with ethidium bromide. Any DNA contamination was removed using TURBO DNase treatment and removal kit from AMBION (Austin, TX, USA).

#### 2.5.2. Real Time Polymerase Chain Reaction (RT-PCR)

From the extracted RNA, cDNA was created by a 20 μL reverse transcriptase (RT) reaction using the BioRad C1000 touch thermocycler using the Improm-II Reverse Transcriptase Kit (Catalog #A1250; Promega, Madison, WI, USA). Firstly, 1 μg of total RNA template, 2 mM of oligo-dT primers, and 10 μM of random hexamer primers were added to the given vial. The optimum annealing temperature was 94 °C for 5 min, copying was 60 min at 42 °C followed by heat inactivation at 70 °C for 15 min. The cDNA obtained was analyzed by Nanodrop (Thermo Fisher Scientific, Waltham, MA, USA) or stored at −80 °C until analysis. The concentration of cDNA was determined by measuring the absorbance at 260 nm and 280 nm with an extinction coefficient of 33 (for single stranded DNA). The extent of genomic DNA contamination was estimated by a RT-PCR assay (real-time) for the reference genes samples.

#### 2.5.3. Primer Design

The primers were designed using Real-Time Primer Design Tool software (IDT DNA, Coralvilla, IA, USA) based on 11 gene sequences from the Genebank database. The primer sequences (17-25-mer), amplicon length (restricted to 90–150 bp) and gene ID can be found summarized in Table 1. The GC content was between 41% and 55%. Primer specificity was verified by BLAST searches against the genomic National Center for Biotechnology Information (NCBI) database. The *Gallus gallus* primer 18S rRNA served as a reference gene.

#### 2.5.4. RT-PCR Design

Performed as described [41]. cDNA (2 μL) was pipetted into a 96-well plate with 2× BioRad SSO Advanced Universal SYBR Green Supermix (8 μL) (Cat #1725274, Hercules, CA, USA); followed by buffer, dNTPs, Taq polymerase and dye. Both forward and reverse primers (as shown in Table 1), cDNA (or water as control) were added to each PCR reaction. Each run had duplicates of 7 standard curve points. A ‘no-template control’ was included with nuclear-free water to detect and exclude any possible DNA contamination. DNA amplification was carried out under the following conditions: initial denaturing at 95 °C for 30 s, 40 cycles of denaturing at 95 °C for 15 s, various annealing temperatures according to Integrated DNA Technologies (IDT) for 30 s and elongating at 60 °C for 30 s in Bio-Rad CFX96 Touch (Hercules, CA, USA). The gene expression data was obtained as the lowest cyclic product (Cp) values based on the automated method of ‘second derivative maximum’. The results were quantified against the standard curve which was diluted at 1:10 and the reaction for each gene were run in duplicates. A graph of Cq vs. log (10) concentrations was produced by the software and the efficiencies were calculated as 10 (1/slope). The specificity of the amplified real-time RT-PCR products was verified by melting curve analysis (60–95 °C) after 40 cycles, resulting in several different specific products, each with a specific melting temperature. Real-time RT-PCR efficiency (E) values for the 11 genes were as follows: DMT-1, 0.998; DcytB, 1.046; Ferroportin, 1.109; ZnT1, 0.954; ZIP1, 0.981; NK-βκ, 1.113; TNF-α, 0.913; IL8, 0.998; SGLT1, 0.994; SI, 1.032; MUC2, 1.022.

### 2.6. Morphological Examination

The intestinal morphology examination was conducted as previously described [37]. The duodenum samples (*n* = 5) collected (Section 2.4) were fixed in fresh 4% (*v*/*v*) formaldehyde solution (stabilized with phosphate buffer), dehydrated, cleaned, and embedded in paraffin. Four sections (5 µm) of each sample were taken and placed on a glass slide. Paraffin was dissolved using xylene, then rehydrated using a series of graded alcohol. This was then stained with Alcian blue/periodic acid-Schiff (PAS) and examined under a light microscope (BX3M series, Olympus Waltham, MA, USA) using the CellSens Standard Software. Paneth cells were stained light purple. The number and diameter of Paneth cells were recorded. Additionally, the villus height, villus width, goblet cell type, diameter and number (in epithelial villi and within circular crypts), and crypt depth were determined for each section. Ten villi per section (4 sections per biological repeat, 5 repeats per treatment group) were measured for statistical analysis.

### 2.7. Cecal Sample Collection and DNA Purification

The cecum samples (*n* = 5) were weighed (0.2 ± 0.02 g) and placed in a 15 mL tube with 9 mL PBS (pH 7.4) under aseptic conditions. Plastic beads (4 mm in diameter) are added to the tube and vortexed for 3 min. The tube was then centrifuged for 5 min at 1000× *g*. The supernatant was collected and centrifuged again at 4000 g for 10 min. After which, the buffer is discarded and the pallet is washed twice with 1 mL PBS. The samples were stored at −20 °C until DNA purification. For the purification, the pellet is treated with 50 mM EDTA (pH 8) and pre-prepared lysozyme (Sigma Aldrich CO., St. Louis, MO, USA) (10 mg/mL) at 37 °C. The DNA isolation was carried out using Wizard Genomic DNA purification kit following the manufacturer’s protocol (Promega Corp., Madison, WI, USA).

### 2.8. Primers Design and PCR Amplification of Bacterial 16S rDNA Analysis

Primers for genus *Clostridium*, *Bifidobacterium*, *Lactobacillus*, and species *E. coli* were designed as per previously published literature [47,48,49]. The primers used in this study are detailed in another study [50]. A universal primer was used to identify all known bacteria and total microflora populations. For the PCR amplification, 5 µL of the purified DNA (from 2.9) was added to 45 µL PCR premixture containing nuclease-free water, dNTPs, PCR buffer, Taq polymerase (Go-Taq; Promega Corporation, Madison, WI, USA), and 10 µg/mL of the primer (repeated for each primer). PCR conditions were set as previously optimized [50]. The PCR products were separated using gel electrophoresis on a 1.5% agarose gel. The gel was stained with light-sensitive ethidium bromide and quantified using Gel-Pro analyzer version 3.0 (Media Cybernetics LP, Rockville, MD, USA). To evaluate the relative portion of each bacterial populations, the products were measured in proportion to the universal primer.

### 2.9. Statistical Analysis

The data in this paper are depicted as their mean values and standard deviation. Experimental treatments and controls for intra-amniotic administration was assigned randomly after ensuring even weight distribution to all groups. ANOVA was used to analyze the results and *p*-values (*p* < 0.05) for significance were determined using post-hoc Duncan test. Software SPSS version 27 (IBM, Armonk, NY, USA) was used for statistical analysis.

## 3. Results

### 3.1. Polyphenol Profile

The relative percentage of the four polyphenols detected in *C. sativus* flower water extract (CFWE) is shown in Table 2 below. There is a high relative abundance of Kaempferol 3-O-sophoroside followed by Kaempferol-3-O-glucoside, Quercetin-3-O-gluside, and Malvidin 3,5-di-O-glucoside in the water extract.

### 3.2. Body Weight and Blood Hemoglobin Concentration

A steady decline in hemoglobin concentration was observed with increase in CFWE concentration with a statistically significant difference between 10% CFWE and the other groups. Although, no significant changes were observed in body weight of the hatchlings, as indicated in Table 3.

### 3.3. Duodenal Gene Expression

**Figure 1:**
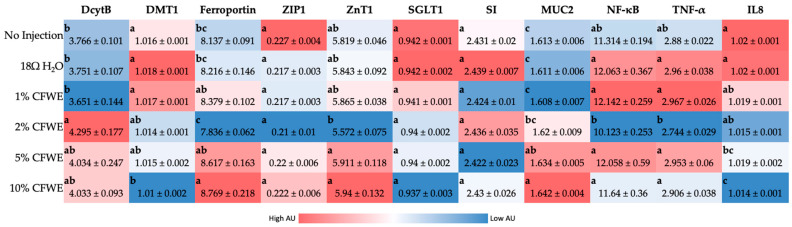
Effect of the intra-amniotic administration of increasing concentration of CFWE and controls on duodenal gene expression. Values are the means (AU: arbitrary units) ± SEM, *n* = 6. ^a, b, c^ genes (column wise) not indicated by the same letter are significantly different (*p* < 0.05). Dcytb, duodenal cytochrome b; DMT1, divalent metal transporter 1; ZIP1, Zrt-, Irt-like proteins; ZnT1, zinc transporter 1; NF-κβ, nuclear factor kappa beta; TNF-α, tumor necrosis factor; IL8, interleukin 8; SGLT1, sodium-glucose cotransporter 1; SI, sucrose isomaltase; MUC2, mucin 2.

Figure 1 indicates the gene expression of proteins related to Zn and Fe metabolism, BBM functionality and immune/inflammatory response.

#### 3.3.1. Fe-Related Protein Gene Expression

Duodenal cytochrome B (Dcytb), ferric reductase enzyme, that reduces Fe^3+^ to Fe^2+^ was upregulated at 2% CFWE (*p* < 0.05). Divalent metal transporter 1 (DMT1) is responsible for the transport of ferrous iron and other divalent mental ions out of the endosomal compartment and/or across the plasma membrane. DMT1 gene expression was significantly downregulated due to 10% CFWE administration. Whereas the gene expression of ferroportin, the protein that exports iron from the cells to the blood was upregulated at 10% CFWE and downregulated at 2% CFWE (*p* < 0.05) [51].

#### 3.3.2. Zn-Related Protein Gene Expression

The gene expression of zinc transporter gene ZIP1 (transports zinc into the cytosol) was not different between the groups. ZnT7 (transports zinc out of the cytosol) expression was reduced (*p* < 0.05) with the intra-amniotic administration of 2% CFWE.

#### 3.3.3. Inflammatory Cytokine Gene Expression

Interleukin 8 (IL8), nuclear factor (NF-κβ), and tumor necrosis factor (TNF-α) are pro-inflammatory genes. NF-κβ and TNF-α expression was downregulated (*p* < 0.05) in case of 2% CFWE treatment. IL8 expression was significantly downregulated with 10% CFWE administration versus controls.

#### 3.3.4. BBM Functionality Related Protein Gene Expression

Mucin 2 (MUC2) are genes that code for mucin proteins that lubricate and protect the intestinal epithelial surface [51]. MUC2 gene expression steadily increased with increase in CFWE concentration (*p* < 0.05). Whereas sucrose isomaltase (SI) and SGLT1 expression were not found to be significantly different among the groups. SI enzyme aids in the hydrolysis of carbohydrate and SGLT1 transports sodium/glucose across the lumen into the enterocyte.

### 3.4. Duodenal Morphological Measurement

#### 3.4.1. *Gallus gallus* Duodenum Cross-Section Images

**Figure 2:**
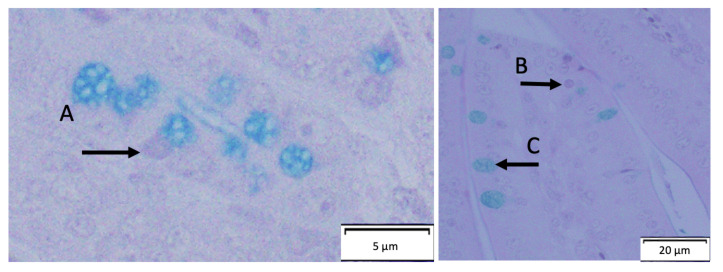
Cross-section of the duodenum (*Gallus gallus*). (**A**) Points out a Paneth cell that is stained light purple. (**B**) Represents a neutral goblet cell, stained light purple. (**C**) Points to an acidic goblet cell, stained bright blue. Stain used: AB/PAS.

Figure 2 are representative images showing a Paneth cell in a circular crypt and goblet cells (acidic and neutral) in a villus.

#### 3.4.2. Goblet Cell Number and Type (Villi and Crypt)

Table 4 indicates that CFWE significantly reduced the goblet cell number in both villi and crypts in comparison to the controls. The decrease is directly proportional to the increase in the concentration of *C. sativus* flower water extract.

#### 3.4.3. Paneth Cell Number, Diameter, and Crypt Depth

As seen in Table 5, the intra-amniotic administration of CFWE resulted in a significant (*p* < 0.05) increase in Paneth cell number and diameter (µM) with maximum values corresponding to the 10% CFWE treatment group. Additionally, it is observed that the crypt depth significantly altered with each treatment without trends.

#### 3.4.4. Average Villi Surface Area and Goblet Cell Diameter

As depicted in Table 6 the villi surface area and goblet cell diameter decreased in the treatment groups when compared to the controls (no injection and H_2_O). A dose-dependent decrease was observed in the two parameters in both the crypt and villi.

### 3.5. Gut Microbiome Analysis

**Figure 3:**
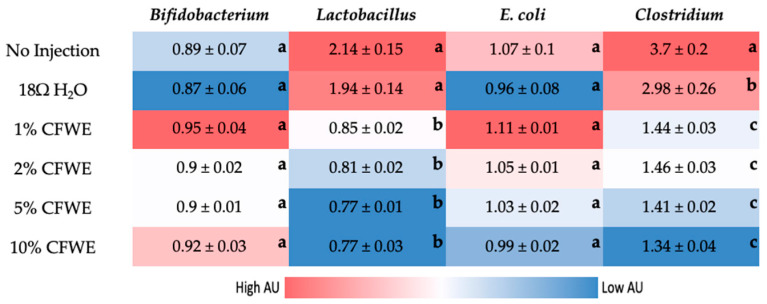
Heatmap showing the effect of different concentrations of dietary CFWE supplementation compared with controls on the populations of *Lactobacillus*, *Clostridium*, *Escherichia coli*, and *Bifidobacterium* in *Gallus gallus* cecum. The relative abundance is expressed in arbitrary units (AU). Values are the means ± SEM, *n* = 5. ^a–c^ groups not indicated by the same letter are significantly different (*p* < 0.05).

The 16s rDNA analysis of cecal bacterial populations showed a significant reduction in relative abundance of genus *Lactobacillus* and *Clostridium* compared to the controls. Although, no statistically significant differences were observed in *Bifidobacterium* and *E. coli* numbers as seen in Figure 3.

## 4. Discussion

The existing literature provides pre-clinical evidence in support of the dietary consumption of *C. sativus* floral bio-residues. However, the data reported in this study demonstrates significant unfavorable changes to the brush border member morphology (BBM), functionality, and microbiome, even at the lowest dose of *C. sativus* water extract (1% CFWE). The cecal microbiome analysis revealed a significant reduction in *Clostridium* and probiotic *Lactobacillus* species. With a rise in dietary products of *C. sativus* flower in the market, it has become essential to assess the effects these foods may have on the intestine, especially in a naïve and developing organism. For the assessment, we utilized the well-established and widely accepted in vivo model (*Gallus gallus*).

From the results, it is seen that CFWE caused a steady dose-dependent decline in hemoglobin (Hb) concentration in the Cornish-cross hatchlings with a significant difference observed at 10% CFWE (*p* < 0.05). These results are in line with previous studies that have shown that certain polyphenols (like quercetin) affect iron absorption in the intestine and hence lower the levels of Hb in the blood [52,53,54,55,56,57]. Some polyphenols are said to chelate iron and hinder its absorption. The reduced Hb concentration (g/dL) result is further supported by the gene expression analysis (Figure 1). The expression of the divalent metal transporter (DMT1) gene was reduced, indicating that there were fewer ferrous iron cations available for absorption/transport. Similarly, the significant upregulation of iron transporter gene, ferroportin (at 10% CFWE), is correlated to the reduced Hb levels observed here. Previous studies have shown that the reduced expression of ferroportin is associated with iron sufficiency [35,58]. We hypothesize that kaempferols and quercetin found in CFWE have resulted in the poorer iron physiological status observed here. Kaempferols found in colored beans (*Phaseolus vulgaris* L.) have previously been shown (in vitro) to inhibit iron absorption [59]. A study by Babaei et al., found no differences in hematological parameter (including Hb) after a 14-day treatment with *C. sativus* petal in rats [60]. This could be as they injected these substances intraperitoneally as opposed to oral consumption hence the chelation of iron in the intestine does not come into question.

In case of the inflammatory biomarkers assessed in the duodenum, it is found that CFWE administration led to a concentration dependent downregulation of the pro-inflammatory gene Interleukin 8 or IL8 (Figure 1). An in vivo study on Wistar rats demonstrated saffron petal hydroalcoholic extract’s inhibitory effects on nuclear factor (NF-κB), C-reactive protein (CRP), and interleukin-6 (IL-6) after 8 weeks [24]. In the present study, however, the expression of NF-κβ and TNF-α genes did not significantly change at 5% and 10% CFWE. This is against our initial hypothesis that saffron floral bio-residue polyphenols would demonstrate an anti-inflammatory effect. Perhaps long-term consumption is required to see the anti-inflammatory effect. The anti-inflammatory properties must be confirmed by further analysis of other inflammation biomarkers and a long-term study.

The results of 16s rDNA analysis showed reduction in two out of the four bacterial populations assessed, indicating a selective antimicrobial effect of the water extract. The polyphenols present in the extract were analyzed and presented in Table 2. Results are in line with existing literature showing a high concentration of kaempferols and malvidin. Two independent studies showed that anthocyanins (from black raspberry) led to a decrease in *Clostridium* and *Lactobacillus* populations in C57BL mice and F-344 rats [61,62]. Several species of the genus *Lactobacillus* are considered probiotic as they can reduce inflammation, improve immune function, and restore homeostasis in many disease conditions [63]. The observed unfavorable changes to the cecal microbiome can be correlated to the changes in brush border membrane (BBM) morphology.

Upon morphometric assessment of the duodenum, there is a significant dose-dependent reduction observed in villi surface area (*p* < 0.05). This observation can be associated with the reduction in Hb levels i.e., reduced mineral absorption capacity of the BBM. Likewise, a significant decrease in villi goblet cell number from 1% CFWE to 10% CFWE is observed. Interestingly, the acidic goblet cell number also reduced drastically from ~33 goblet cells per villi in the (H_2_O control group) to ~23 goblet cells per villi (in 10% CFWE group). A reduction in acidic goblet cells indicates a reduced potential for an acidic BBM environment. A lower luminal pH supports the growth of health promoting bacteria [40,46]. This reduction in acidic goblet cells can be correlated to the reduction observed in the *Lactobacillus* populations (Figure 3). In addition, the diameter of the goblet cell was also found to be significantly reduced with increasing concentration of CFWE. This indicates a decreased mucin secreting capacity which is crucial for the protection of the intestinal barrier, the commensal microbiome, and overall BBM functionality. With the reduction in the goblet cells, it is up to the Paneth cells to regulate the immune function and protect the intestinal barrier. This is seen with the increase in Paneth cell number and diameter with concentration. We hypothesize that the reduction in mucin production due to lower goblet cell number and diameter led to an increase in Paneth cell number and diameter. Previously, an increase in Paneth cell number has been shown to be associated with toxicity and infection [64,65]. There is also a significant increase in MUC2 gene (*p* < 0.05) to compensate for the reduced goblet cell numbers with increasing CFWE concentrations. The expression of the MUC2 gene has been shown to increase with quercetin [66].

## 5. Conclusions

The present study demonstrates that the dietary consumption of *C. sativus* flower water extract leads to a reduction in villi surface area, decrease in goblet cell (number and diameter), reduced hemoglobin levels and unfavorable changes to the microbiome in-vivo (*Gallus gallus*). Based on these findings, it is not recommended for infants and individuals suffering from iron deficiency to consume products containing saffron flowers. Although further studies are required to support this interpretation. This is a preliminary study to get an impression of the potential effects the extract may have on intestinal health.

## Figures and Tables

**Table 1:** DNA sequences of primers used.

Analyte	Forward Primer (5′→3′)	Reverse Primer (5′→3′)	Base Pair	GI Identifier
Iron Metabolism				
DcytB	CATGTGCATTCTCTTCCAAAGTC	CTCCTTGGTGACCGCATTAT	103	20,380,692
DMT1	TTGATTCAGAGCCTCCCATTAG	GCGAGGAGTAGGCTTGTATTT	101	206,597,489
Ferroportin	CTCAGCAATCACTGGCATCA	ACTGGGCAACTCCAGAAATAAG	98	61,098,365
Zinc Metabolism				
ZIP1	TGCCTCAGTTTCCCTCAC	GGCTCTTAAGGGCACTTCT	144	107,055,139
ZnT1	GGTAACAGAGCTGCCTTAACT	GGTAACAGAGCTGCCTTAACT	105	54,109,718
Inflammatory Response				
NF-κβ	CACAGCTGGAGGGAAGTAAAT	TTGAGTAAGGAAGTGAGGTTGAG	100	2,130,627
TNF-α	GACAGCCTATGCCAACAAGTA	TTACAGGAAGGGCAACTCATC	109	53,854,909
IL8	TCATCCATCCCAAGTTCATTCA	GACACACTTCTCTGCCATCTT	105	395,872
BBM functionality				
SGLT1	GCATCCTTACTCTGTGGTACTG	TATCCGCACATCACACATCC	106	8,346,783
SI	CCAGCAATGCCAGCATATTG	CGGTTTCTCCTTACCACTTCTT	95	2,246,388
MUC2	CCTGCTGCAAGGAAGTAGAA	GGAAGATCAGAGTGGTGCATAG	155	423,101

Dcytb, duodenal cytochrome b; DMT1, divalent metal transporter 1; ZIP1, Zrt-, Irt-like proteins; ZnT1, zinc transporter 1; NF- κβ, nuclear factor kappa beta; TNF-α, tumor necrosis factor; IL8, interleukin 8; SGLT1, sodium-glucose cotransporter 1; SI, sucrose isomaltase; MUC2, mucin 2.

**Table 2:** Concentration of polyphenols in the CFWE prepared.

Polyphenolic Compounds	% *
Malvidin 3,5-di-O-glucoside	0.03
Kaempferol-3-O-glucoside	7.90
Quercetin-3-O-gluside	0.37
Kaempferol-3-O-sophoroside	91.70

* Table shows % polyphenols from total assessed.

**Table 3:** Average hemoglobin and body weight in all groups.

Treatment Group	Average Hemoglobin (g/dL)	Average Body Weight (g)
No Injection	12.52 ± 0.91 ^a^	42.06 ± 1.35 ^a^
18 Ω H_2_O	10.13 ± 0.71 ^a^^,^^b^	42.16 ± 1.28 ^a^
1% CFWE	12.26 ± 0.72 ^a^	42.96 ± 0.91 ^a^
2% CFWE	11.15 ± 0.56 ^a^	42.13 ± 0.68 ^a^
5% CFWE	10.24 ± 0.73 ^a^	42.89 ± 0.77 ^a^
10% CFWE	9.62 ± 1.21 ^b^	42.88 ± 0.96 ^a^

Values are means ± SEM, *n* = 10. ^a, b^ Treatment groups not indicated by the same letter are significantly different (*p* < 0.05).

**Table 4:** Effect of intra-amniotic administration of investigated concentration of CFWE and controls on goblet cell type and total number of goblet cells in duodenal villi and crypts.

Treatment Group	Average Goblet Cell Number Per Villi			Total Villi Goblet Number	Total Crypt Goblet Number
	Acidic	Neutral	Mixture		
No Injection	36.76 ± 0.85 ^a^	0.02 ± 0.01 ^a^	0.3 ± 0.06 ^a^	37.08 ± 0.86 ^a^	8.33 ± 0.25 ^a^
18 Ω H_2_O	32.92 ± 0.68 ^b^	0 ± 0 ^a^	0.05 ± 0.03 ^b^	32.97 ± 0.68 ^b^	7.44 ± 0.22 ^b^
1% CFWE	29.28 ± 0.86 ^c^	0.03 ± 0.02 ^a^	0.04 ± 0.02 ^a^	29.34 ± 0.87 ^c^	6.73 ± 0.22 ^c^
10% CFWE	22.49 ± 0.5 ^d^	0.01 ± 0.01 ^a^	0.01 ± 0.01 ^b^	22.51 ± 0.5 ^d^	6.68 ± 0.22 ^c^

Values are the means ± SEM, *n* = 5. ^a–d^ Treatment groups not indicated by the same letter are significantly different (*p* < 0.05).

**Table 5:** Effect of intra-amniotic administration of different concentration of CFWE on Paneth cell number and diameter.

Treatment Group	Paneth Cell Number	Paneth Cell Diameter (µM)	Crypt Depth (µM)
No Injection	2.24 ± 0.08 ^b^	1.56 ± 0.03 ^c^	45.77 ± 1.32 ^b^
18 Ω H_2_O	1.89 ± 0.07 ^c^	1.89 ± 0.05 ^b^	50.73 ± 1.1 ^a^
1% CFWE	1.93 ± 0.07 ^c^	1.67 ± 0.03 ^c^	32.81 ± 0.73 ^d^
10% CFWE	2.56 ± 0.08 ^a^	2.47 ± 0.06 ^a^	37.75 ± 0.8 ^c^

Values are the means ± SEM, *n* = 5. ^a–d^ Treatment groups not indicated by the same letter are significantly different (*p* < 0.05).

**Table 6:** Effect of intra-amniotic administration of treatments and controls on villi surface area and goblet cell diameter in duodenal villi and crypts.

Treatment Group	Average Surface Area (mm^2^)	Villi Goblet Cell Diameter (µM)	Crypt Goblet Cell Diameter (µM)
No Injection	168.19 ± 3.72 ^a^	4.65 ± 0.06 ^b^	2.98 ± 0.06 ^c^
18 Ω H_2_O	171.45 ± 4.2 ^a^	5.13 ± 0.06 ^a^	3.32 ± 0.07 ^a^
1% CFWE	137.91 ± 3.4 ^b^	4.18 ± 0.06 ^c^	3.11 ± 0.06 ^b^^,^^c^
10% CFWE	116.41 ± 2.71 ^c^	3.95 ± 0.06 ^d^	3.17 ± 0.06 ^a^^,^^b^

Values are the means ± SEM, *n* = 5. ^a–d^ groups not indicated by the same letter are significantly different (*p* < 0.05).
